# Comparison of variations between percentage of body fat, body mass index and daily physical activity among young Japanese and Thai female students

**DOI:** 10.1186/1880-6805-31-21

**Published:** 2012-08-15

**Authors:** Tomoko Morinaka, Porn-ngarm Limtrakul, Luksana Makonkawkeyoon, Yoshiaki Sone

**Affiliations:** 1 Graduate School of Human Life Science, Osaka City University, Sugimoto, Sumiyoshi-ku, Osaka, Japan; 2 Department of Biochemistry, Faculty of Medicine, Chiang Mai University, Chiang Mai, Thailand

**Keywords:** Body mass index, Body fat percentage, Physical activity, Uniaxial accelerometer

## Abstract

**Background:**

In our series of investigations concerning the causes of seasonal change in fat accumulation in young university students, we could not find any contribution of seasonal variation in the ratio of carbohydrate and fat metabolism to that of body fat percentage in Japanese and Thai participants. After our previous study, we examined the effect of daily physical activity on body fat percentage to look for the major causes of seasonal change in fat accumulation in young university students.

**Findings:**

In this study, we measured participants’ (young Japanese and Thai university students) daily physical activity by a uniaxial accelerometer in addition to the measurements of body fat percentage and body mass index by a bioelectrical impedance meter. We found that there was significant and moderate negative correlation between body fat percentage and daily step counts among Japanese but not Thai participants. We observed significant, moderate and positive correlations between the percentage of body fat and body mass index among Japanese and Thai participants.

**Conclusions:**

Daily physical activity plays an important role in the seasonal variation of body fat percentage of Japanese female students. Our present study also confirmed the importance of daily physical activity for controlling body mass index and for the prevention of obesity.

## Background

We are interested in what is behind obesity from the point of view of human nutritional evolution, such as the ‘mismatch’ of our present nutritional physiology with the foods we eat today and the levels of energy we expend in our daily lives [1]. During the course of our investigation in this regard, we researched the causes of seasonal change in body fat percentage and body mass index on the assumption that seasonal changes in the balance of carbohydrate and fat metabolism is an intrinsic physiological mechanism for the seasonality of body fat percentage [2]. In previous research, we could not find any significant correlation between the seasonal changes in the balance of carbohydrate/fat metabolism and the percentage of body fat among Japanese, Polish and Thai participants, suggesting that the effect of such intrinsic factors has become so small that it no longer has so much influence on body fat percentage. Concerning the factors that affect body fat percentage, Buchowski *et al*. [3] and den Hoed *et al*. [4] reported a negative association between body fat percentage and daily physical activity measured by a triaxial accelerometer. These reports prompted us to examine the variations between body fat percentage, body mass index and daily physical activity among the participants by measuring these factors several times a year with Japanese and Thai students, who belonged to the same group as those who had participated in our previous research. The aim of this study is to show the effect of daily physical activity on the seasonal change in the body fat percentage and body mass index of Japanese and Thai female university students.

## Participants and methods

### Participants

Twenty-two Japanese and twenty-seven Thai female university students participated in this study as paid participants. All participants were non-smokers and healthy, according to their self-assessment. The participants were female university students attending the universities of the authors (Osaka City University in Japan, and Chiang Mai University in Thailand) and their general description is shown in Table 1. Comparison of the items listed in Table 1 between these two sample groups shows there were no significant differences except for the measured percentage of body fat (t (47) = 2.300, *P* = 0.026). We explained the purpose of the study and the procedures involved to all the participants before they gave their written consent to participate according to the protocol approved by the Research Ethics Committee of the Graduate School of Human Life Science, Osaka City University (Approval No. 08/01) and the Research Ethics Committee of the Faculty of Medicine, Chiang Mai University (Approval No. Bio-11-02-21-12-X).

**Table 1:** Characteristics of the participants

	**Japanese (*n*22)**	**Thai (*n*27)**
	**Mean ± SD (range)**	**Mean ± SD (range)**
Age (years)	21.5 ± 0.7 (21–24)	21.2 ± 2.2 (18–25)
Height (cm)	159.9 ± 5.9 (151.7-170.5)	160.6 ± 6.0 (146.0-170.0)
Body fat (%)	23.0 ± 5.3^a^ (12.2-35.4)	26.7 ± 5.6^a^ (15.8-36.1)
Weight (kg)	49.5 ± 4.2 (43.6-58.3)	51.2 ± 5.9 (41.4-64.6)
Body mass index (kg/m^2^)	19.4 ± 1.5 (16.5-22.1)	19.8 ± 2.0 (16.6-23.0)

The values are mean, standard deviation, and range of characteristics. These values were obtained when the examination started, in May 2010 for Japan and Apr 2011 for Thailand. ^a^Means sharing a common superscript letter are significantly different at *P* <0.05 by Student’s *t*-test.

### Measurements

The participants’ body fat percentage, body weight and body mass index were measured and calculated by a segmental multifrequency bioelectrical impedance meter (InBody430, Biospace Japan Inc., Tokyo, Japan) in both samples. Measurements were carried out in the morning after an overnight fast wearing the same toweling dress of 0.5 kg throughout the examination (with Japanese participants) or without their outerwear (with Thai participants). The height of each participant was measured to the nearest 0.1 cm by a usual scale. Daily physical activity was measured on seven consecutive days before the day of body composition measurement by means of a uniaxial accelerometer (Lifecorder EX, Suzuken, Nagoya, Japan), which was attached to the participant’s waist from the time they woke up until they went to sleep. The daily step count of the participants stored in the accelerometer was computed by its software for the analysis (Lifelyzer 02 Pro, Suzuken, Nagoya, Japan). In Japan, we measured body composition every month from May (2010) to January (2011) and monitored daily physical activity in May, July, October (2010) and January (2011). In Thailand, body composition and daily physical activity were measured in April, June, August, October, December (2011) and January (2012). Hereafter, each month the measurements were carried out will be abbreviated to three characters.

### Data analyses

Changes in the variable through the measuring periods (temporal change) were analyzed by one factor (measuring time) repeated measure analysis of the variance (ANOVA) followed by post hoc multiple comparison tests using the Bonferroni method. Multiple regression was used to determine the variations between the variables such as the body fat percentage, body mass index, and number of steps per day according to the reference ‘Calculating correlation coefficients with repeated observations: Part 1 - correlation within subjects’ of Bland and Altman [5]. The *P* value < 0.05 (two-sided) was considered to be statistically significant. These analyses were performed using the Statistical Package for the Social Sciences version 16.0 (IBM SPSS, Chicago, IL, USA).

## Results

Table 2 shows the temporal changes in the body fat percentage, body mass index and daily step counts of the two sample groups. One factor repeated ANOVA showed significant changes in the percentage of body fat, body mass index and daily step counts of Japanese participants (*F* (8, 168) = 9.741, *P* = 0.000, *F* (8, 168) = 6.533, *P* = 0.000, *F* (3, 63) = 4.138, *P* = 0.010, respectively). In Thai participants, there were no significant seasonal changes in the percentage of body fat or body mass index, (*F* (5, 130) = 0.826, *P* = 0.533, *F* (5, 130) = 0.109, *P* = 0.990, respectively). There was, however, a significant seasonal change in daily step counts (*F* (5, 130) = 6.043, *P* = 0.000). Table 3 summarizes the results of the multiple regression analyses of the variables measured in this study. Between the body fat percentage and daily step counts, significant and moderate negative correlation was found among Japanese participants, but not among Thai participants. We observed significant, moderate and positive correlations between the percentage of body fat and body mass index among Japanese and Thai participants.

**Table 2:** Summary of the mean and standard deviations of the measurements

**Subjects**			
**Month**	**Body fat %**	**Body mass index**	**Daily step counts**
**Japanese (n = 22)**			
Apr			
May	23.0 ± 5.3	19.4 ± 1.5^a^	10329 ± 3208
Jun	22.2 ± 4.9^a,b^	19.2 ± 1.6	
Jul	22.0 ± 5.0^c,d,e^	19.1 ± 1.6	10493 ± 4128
Aug	22.1 ± 5.0^f,g,h^	19.0 ± 1.5^b,c^	
Sep	22.4 ± 5.2^i,j^	19.0 ± 1.5^a,d,e,f^	
Oct	23.7 ± 4.8^a,c,f,i^	19.1 ± 1.6	9514 ± 2991
Nov	23.7 ± 4.7^b,d,g,j^	19.3 ± 1.5^b,d^	
Dec	23.4 ± 4.9	19.3 ± 1.6^e^	8466 ± 2876
Jan	24.0 ± 4.9^e,h^	19.4 ± 1.6^c,f^	
Year^*^			9701 ± 3377
**Thai (n = 27)**			
Apr	26.7 ± 5.6	19.8 ± 2.0	6977 ± 2560
May			
Jun	26.3 ± 5.7	19.9 ± 2.2	7434 ± 2242^a,b^
Jul			
Aug	27.0 ± 6.1	20.0 ± 2.2	6132 ± 1972^a^
Sep			
Oct	27.2 ± 6.0	20.0 ± 2.3	5375 ± 2448^b^
Nov			
Dec	27.7 ± 5.8	20.1 ± 2.4	6237 ± 2110
Jan	28.1 ± 6.8	19.9 ± 2.3	6909 ± 2218
Year^*^			6511 ± 2332

^a-j^Means in the same column sharing a common superscript letter are significantly different, *P* <0.05. ^*^Comparison of pooled data. Significantly different by *t*-test (t = 7.897, *P* = 0.000).

**Table 3:** Bivariate correlation of body fat percentage, body mass index, and daily step counts

**Subjects**	**Dependent variable**	**Correlation coefficient within subjects**	***P***
**Independent variable**			
**Japanese (n = 22)**			
Step counts per day	Body fat %	−0.480	0.000
Body fat %	Body mass index	0.443	0.000
Step counts per day	Body mass index	−0.007	0.952
**Thai (n = 27)**			
Step counts per day	Body fat %	−0.074	0.393
Body fat %	Body mass index	0.791	0.000
Step counts per day	Body mass index	−0.067	0.441

## Discussion

In this study, we adopted steps taken per day as an indicator of the daily physical activity of Japanese and Thai participants because Swartz *et al*. [6] reported that steps taken per day recorded by Lifecorder EX provided more beneficial and reliable feedback on physical activity level, rather than kilocalories calculated based on the thermic effect of food, physical activity energy expenditure, and an estimation of basal metabolic rate developed on Japanese participants. In terms of the main purpose of this study, to show the effect of daily physical activity on the seasonal changes in the percentage of body fat and body mass index of the Japanese and Thai female university students, we obtained different results among the two samples. In Japanese participants, there was a significant temporal change in the percentage of body fat, whose changing pattern was the same as that observed in our previous study [2]. As shown in Table 3, a significant and negative association between body fat percentage and the steps per day was observed among Japanese participants. Considering no significant correlation between the energy intake (kcal/day) and the percentage of body fat among Japanese participants observed in our previous study [2], the converse relationship between the temporal changing pattern of the step counts and that of the body fat percentage, and the significant negative correlation between them observed in this study, may indicate that daily physical activity plays an important role in the seasonal variation of body fat percentage observed in our previous and the present study for Japanese female students.

In contrast to the results of the Japanese participants, there was no significant seasonal change in the percentage of body fat or correlation between the percentage of body fat and the steps per day among Thai participants. No significant correlation between the body fat percentage and the steps per day may be explained by less daily physical activity as well as the shorter time or strength of physical activity than that of Japanese participants; den Hoed and Westerterp reported that body fat percentage was negatively associated with total daily physical activities, especially with the length of time for physical activities of higher intensity [4]. When we compared the data collected by Lifecorder EX, the mean number of steps of Thai students through the observation period was significantly lower than that of Japanese students (see Table 2). This low daily physical activity may be a major factor for no seasonal change in the percentage of body fat of Thai participants. The lesser amount of daily physical activity of Thai students compared to that of Japanese students could be explained by the difference in their lifestyle. Thai co-authors reported that most of Thai participants were living in the university dormitories and some were living in houses in the town of Chiang Mai, usually using motorcycles or cars to travel to the university. Concerning the relationship between the daily step counts and the transportation means of the university students, Oie *et al*. [7] reported, though about the Japanese students, that the students living in a rural area who usually use their car to commute tended to take fewer steps than those living in an urban area.

In addition to the difference in daily physical activity between the two samples described above, difference in the climate change between the two cities could not be ignored as a factor influencing the seasonal change in daily physical activity (hence in their percentage of body fat) of the two sample groups. As mentioned in our previous paper [8], Thailand has a warm, tropical climate affected by an annual monsoon and the annual average temperature in Chiang Mai is around 26°C and the mean monthly temperature is relatively constant when compared to that of Japan (the difference in the temperature is less than 8°C in Chiang Mai, in contrast to more than 20°C in Osaka). This constancy of the air temperature in Chiang Mai may contribute to less seasonal change in the daily physical activity compared to that of Japanese participants because a change in climate change influences daily physical activity [9].

From the point of view of obesity prevention, in this study we found significant and positive correlation between the percentage of body fat and body mass index among both Japanese and Thai participants. On this topic, several studies have also reported the positive relationship between body fat percentage and body mass index but the relationship is ethnic-specific [10]. Of course, we should consider the contribution of lean body weight to body weight (and body mass index, [11]); our present study confirmed the importance of daily physical activity for controlling body mass index and for the prevention of obesity.

## Abbreviation

ANOVA: analysis of the variance.

## Competing interests

The authors declare that they have no competing interests.

## Authors’ contributions

YS was a general coordinator and did the study design. PL and LM designed the study and involved in data collection, data interpretation and result analysis. TM were involved in data collection, data interpretation, and result analysis and literature search. All authors contributed in preparing and read and approved the final manuscript.
